# Recruitment and retention in a preclinical AD trial: comparisons between academic and non-academic sites

**DOI:** 10.1186/s13195-025-01867-8

**Published:** 2025-10-14

**Authors:** Marina Ritchie, Kedir Hussen, Oliver Langford, Christian Navarro, Zara Kotadiya, Michael C. Donohue, Paul Aisen, Reisa A. Sperling, Joshua D. Grill, Rema Raman

**Affiliations:** 1 Alzheimer’s Therapeutic Research Institute, University of Southern California, San Diego, CA USA; 2 University of California, Santa Barbara, Santa Barbara, CA USA; 3 University of Virginia, Charlottesville, VA USA; 4 Center for Alzheimer Research and Treatment, Brigham and Women’s Hospital, Harvard Medical School, Boston, MA USA; 5 University of California, Irvine, Irvine, CA USA

**Keywords:** Site type, Clinical trials, Retention, Recruitment, Preclinical alzheimer’s disease

## Abstract

**Background:**

Alzheimer’s disease (AD) clinical trials enroll participants at various site types including research-focused academic institutions and independent non-academic sites. Limited research has examined the impact of site type on recruitment and retention outcomes.

**Methods:**

To evaluate potential differences between site types, we used data from the Anti-Amyloid Treatment for Asymptomatic AD (A4) trial, the largest completed preclinical AD trial to date. We first compared the frequency of varying recruitment sources between site types. We then examined potential differences in participant- and site-level characteristics. To assess potential site type differences in retention, we fit a multivariable logistic regression model adjusting for variables associated with site type. For participants who prematurely discontinued, we examined potential differences by site type in reasons for dropout.

**Results:**

One thousand and fifty-eight participants were randomized at 50 academic (*N* = 835) and 15 non-academic (*N* = 223) sites in North America. Academic sites had higher proportions of participants recruited through earned media and organizational referrals and lower proportions recruited through internal referrals and advertisements, compared to non-academic sites. Participant-level characteristics differed between site types. Compared to non-academic sites, academic sites had higher proportions of participants with a family history of dementia and a professional degree (highest education category), but lower proportions of individuals with a history of diabetes, a CDR-SB score above 0, and belonging to a racial and ethnic underrepresented group. Though the results were not statistically significant, non-academic sites had a higher screening rate (number of participants screened/site/month), but a lower randomization rate (randomized/screened) compared to academic sites. No site type differences in completion rates were observed. When examining reasons for discontinuation, we found that among the 72 participants who discontinued the trial at non-academic sites, 56 (77.8%) withdrew consent or were lost to follow up. In contrast, 140 out of 243 (57.6%) participants who discontinued the trial in academic sites withdrew consent or were lost to follow up.

**Conclusion:**

Our findings shed light on important site type differences that investigators should consider when making choices around site, design, and conduct in multisite preclinical AD trials.

**Supplementary Information:**

The online version contains supplementary material available at 10.1186/s13195-025-01867-8.

## Background

Large phase II and III clinical trials often enroll participants through multiple sites to accelerate accrual and enhance trial efficiency [1]. While all sites follow a single protocol, it is unclear whether systematic differences in the conduct of trials exist among site types that could impact participant recruitment and retention outcomes; two determinative, but largely understudied, factors of clinical trial success [2–7].

In multisite symptomatic Alzheimer’s disease (AD) trials, sites fail to enroll one participant per month on average [8] and discontinuation rates, particularly for longer duration trials, often exceed expectations [9]. Recently, as the field has moved toward targeting earlier stages of disease, cognitively unimpaired participants have been enrolled in preclinical AD trials aimed at delaying symptom onset. All participants in these trials are required to undergo biomarker testing such as positron emission tomography (PET) imaging, as well as other imaging, blood draws, and extensive cognitive testing [10]. While there is increasing literature suggesting that undergoing biomarker testing and learning results is not a barrier to participation [11, 12], these trials face novel challenges to recruitment and retention as they are typically longer in duration, have high rates of ineligibility, and require larger sample sizes to achieve sufficient power [13, 14].

The network of sites necessary to conduct AD trials frequently includes academic institutions as well as non-academic sites such as independent clinic/hospital centers and commercial research organizations. Data from one mild cognitive impairment (MCI) trial suggested that NIH-funded academic Alzheimer’s Disease Research Centers (ADRCs) were almost two-fold more likely to retain participants to study completion than non-academic sites [15]. Identifying such site type differences in a preclinical AD trial may guide the development and implementation of more targeted retention strategies in future trials.

In a prior analysis of the Anti-Amyloid Treatment for Asymptomatic AD (A4) trial, one of the first and largest multi-center completed preclinical AD trials to date [10], characteristics such as baseline anxiety score and age were associated with early trial discontinuation, while being an APOE e4 carrier and having a family history of AD were associated with higher completion rates [16]. Here, we sought to better understand whether participant- and site-level characteristics, as well as recruitment sources, retention rates, and reasons for discontinuation, differed between academic and non-academic sites in the A4 trial.

## Methods

### Data source

We used data from the A4 trial (ClinicalTrials.gov identifier: NCT02008357; registration date: December 6th, 2013), a multi-center preclinical AD trial testing the efficacy and safety of solanezumab vs. placebo over 240 weeks [10]. Participants in A4 were cognitively unimpaired, ages 65–85, and had elevated brain levels of beta-amyloid as detected by ^18^F-florbetapir PET. All A4 participants reviewed and signed an IRB-approved consent form before enrollment. We used deidentified data from the screening phase for our analysis on recruitment sources. For all other analyses, we used the double-blind phase of the randomized controlled trial.

### Derived variables

*Site types*. For the purposes of these analyses, we defined *site type* as academic (universities and university-affiliated hospitals) or non-academic (independent clinics/institutions and private hospitals).

*Participant characteristics*. Given the small number of individuals who self-identified as being non-White race or Hispanic ethnicity (*N* = 76), we created a combined *racial and ethnic underrepresented group (RE-URG)* variable as follows: participants who self-reported as being of American Indian or Alaska Native race, Asian race, Black or African American race, more than one race, or of Hispanic ethnicity were included in the RE-URG category. Individuals who self-reported as being of White race and not of Hispanic ethnicity were considered not a RE-URG.

*Site characteristics*. We defined *screening rate* as the number of screened participants per screening month per site. The screening months reflect the duration (in months) between the first screened participant at the site and the last randomized participant in the trial. The *randomization rate* is defined as the number of randomized participants divided by the total number of screened participants at the site-level.

*Recruitment sources* were defined a priori as (a) ‘advertising,’ which included paid advertisement, (b) ‘earned media’ in the form of news or other non-paid content, (c) ‘internal referral’ from site staff and databases, (d) ‘organizational referral’ such as the Alzheimer’s Association, National Institutes of Health (NIH), American Association of Retired Persons (AARP), and (e) ‘outside physician referral’. Participants had the option of selecting more than one recruitment source.

*Primary reasons for discontinuation* were thematically categorized as ‘adverse events’ (i.e., death; safety risk; adverse event), ‘participant withdrawal or lost to follow-up’ (i.e., perceived lack of efficacy; non-compliance; participant unwilling or unable to participate; study partner unwilling or unable to participate; lost to follow up), or ‘other’ (i.e., investigator recommendation; other non-site clinician recommendation; coordinating center request; started prohibited medication; starting new trial; covid-19 pandemic disruption; starting a new treatment for AD; other).

### Statistical analyses

We utilized screening and follow-up data on randomized participants enrolled in one of 65 North American sites. We excluded data for participants enrolled in Japan and Australia (one academic site each) given the lack of variability in site types as well as the potential cultural and regional differences. Relevant participant- and site-level characteristics were reported using means and standard deviations for continuous variables and frequencies along with percentages for categorical variables. We examined potential differences by site type in recruitment sources using a chi-square test with Holm’s adjustments to account for multiple comparisons for recruitment source. To evaluate whether site type was associated with trial completion, we first examined potential differences in time-to-dropout by using a Kaplan-Meier estimator and log-rank test. We a priori determined to proceed with a time-to-event model for our subsequent multivariable analysis if the time to dropout event was associated with site type; else, we would use a simpler binary outcome model. To investigate potential differences in participant- and site-level characteristics, we conducted a series of univariate analyses using a chi-square test and Fisher’s exact test, as appropriate, for categorical variables, and a two-sample t-test for continuous variables. We fit a multivariable logistic mixed-effect regression model with participant-level completion status as the binary outcome, site-specific random intercepts, and fixed effects for variables associated with site type in the univariate analyses at the *p* < 0.100 level. For participants who prematurely discontinued the trial, we examined the frequency with which they reported different reasons for early discontinuation and evaluated whether these reasons differed by site type using a chi-square test. As further exploratory analyses, we examined the subcategories of those who discontinued due to adverse events and the baseline participant characteristics of those who discontinued due to voluntary withdrawal or lost to follow-up by site type to identify any variables that may be associated with voluntary withdrawal. All statistical analyses were conducted using the statistical software R version 4.2.2.

## Results

One thousand and fifty-eight participants in North America were randomized from 50 academic (*N* = 835) and 15 non-academic (*N* = 223) sites. Data from 835 participants (78.9%) in academic sites and 223 participants (21.1%) in non-academic sites were analyzed in this study. The mean (SD) number of participants randomized was 16.7 (12.9) for academic sites and 14.9 (14.0) for non-academic sites. The mean participant age was 72 years, and the majority of participants were female, highly educated, married, had a family history of dementia, and identified as being non-Hispanic ethnicity and White race.

*Participant and site characteristics*. Table 1 presents participant characteristics for the site types. We observed a higher proportion of participants belonging to a RE-URG (academic: 6.3%; non-academic: 11.0%; *p* < 0.001) and a lower proportion of individuals with a professional degree (highest education category) (academic: 47.1%: non-academic: 30.9%; *p* < 0.001) among participants enrolled at non-academic sites. Academic sites had a higher proportion of participants who enrolled with a spousal study partner (academic: 65.9%; non-academic: 60.5%; *p* = 0.023) and had a known family history of dementia (academic: 77.1%; non-academic: 69.5%; *p* = 0.019). Clinical characteristics of participants at baseline were comparable across site type except for the CDR-SB, with a higher proportion of participants scoring above 0 in non-academic sites (academic: 7.8%; non-academic: 23.3%; *p* < 0.001). Medical history of diabetes was also more common among participants at non-academic sites (academic: 7.9% non-academic: 13.0%; *p* = 0.018).

**Table 1:** Differences in baseline participant characteristics by site type and the corresponding *p*-values

	Academic (*N* = 835)	Non-academic (*N* = 223)	Total (*N* = 1058)	Unadj. *P* Value
Sex Female, *n* (%)	501 (60.0%)	135 (60.5%)	636 (60.1%)	0.884^1^
Age at Consent, Mean (SD)	72.0 (4.8)	71.7 (5.0)	72.0 (4.8)	0.353^2^
Race				NA
Missing, *n*	5	2	7	
American Indian or Alaskan Native, *n* (%)	0 (0.0%)	2 (0.9%)	2 (0.2%)	
Asian, *n* (%)	6 (0.7%)	1 (0.5%)	7 (0.7%)	
Black or African American, *n* (%)	20 (2.4%)	8 (3.6%)	28 (2.7%)	
Native Hawaiian or Other Pacific Islander, *n* (%)	0 (0.0%)	0 (0.0%)	0 (0.0%)	
More than one race, *n* (%)	6 (0.7%)	2 (0.9%)	8 (0.8%)	
White, *n* (%)	798 (96.1%)	208 (94.1%)	1006 (95.7%)	
Ethnicity				
Missing, *n*	7	4	11	NA
Hispanic or Latino, *n* (%)	21 (2.5%)	12 (5.5%)	33 (3.2%)	
Race and Ethnicity URG				0.018^1^
Missing, *n*	8	4	12	
URG, *n* (%)	52 (6.3%)	24 (11.0%)	76 (7.3%)	
Marital Status				0.154^1^
Missing, *n*	10	1	11	
Divorced, *n* (%)	112 (13.6%)	42 (18.9%)	154 (14.7%)	
Never married, *n* (%)	35 (4.2%)	6 (2.7%)	41 (3.9%)	
Widowed, *n* (%)	75 (9.1%)	16 (7.2%)	91 (8.7%)	
Married, *n* (%)	603 (73.1%)	158 (71.2%)	761 (72.7%)	
Study Partner Type				0.023^1^
Missing, *n*	17	0	17	
Spouse, *n* (%)	539 (65.9%)	135 (60.5%)	674 (64.7%)	
Adult Child/Child-in-law, *n* (%)	106 (13.0%)	22 (9.9%)	128 (12.3%)	
Other, *n* (%)	173 (21.1%)	66 (29.6%)	239 (23.0%)	
Family Dementia History, *n* (%)	644 (77.1%)	155 (69.5%)	799 (75.5%)	0/019^1^
Education Category				< 0.001^1^
High School Graduate and Below, *n* (%)	60 (7.2%)	26 (11.7%)	86 (8.1%)	
Some College Degree, *n* (%)	382 (45.7%)	128 (57.4%)	510 (48.2%)	
Professional Degree, *n* (%)	393 (47.1%)	69 (30.9%)	462 (43.7%)	
Treatment Group				0.908^1^
Placebo, *n* (%)	423 (50.7%)	112 (50.2%)	535 (50.6%)	
Solanezumab, *n* (%)	412 (49.3%)	111 (49.8)	523 (49.4%)	
Baseline Mini-Mental State Examination				0.285^2^
Missing, *n*	4	0	4	
Mean (SD)	28.8 (1.2)	28.7 (1.3)	28.8 (1.2)	
Baseline PACC, Mean (SD)	0.1 (2.7)	0.0 (2.6)	0.0 (2.7)	0.787^2^
Baseline Geriatric Depression Scale				0.304^2^
Missing	5	1	6	
Mean (SD)	1.0 (1.3)	1.1 (1.3)	1.0 (1.3)	
Baseline State-Trait Anxiety Inventory				0.188^2^
Missing	5	1	6	
Mean (SD)	9.9 (3.1)	9.6 (2.8)	9.8 (3.0)	
Baseline Impact of Event Scale				0.188^2^
Missing	8	1	9	
Mean (SD)	10.0 (10.6)	11.1 (11.5)	10.2 (10.8)	
Baseline CFI Participant, Mean (SD)	2.3 (2.1)	2.4 (2.3)	2.3 (2.2)	0.457^2^
Baseline CDR Sum of Box Category				< 0.001^1^
0	770 (92.2%)	171 (76.7%)	941 (88.9%)	
Above 0	65 (7.8%)	52 (23.3%)	117 (11.1%)	
Diabetes Medical History	66 (7.9%)	29 (13.0%)	95 (9.0%)	0.018^1^
Baseline Framingham Score				0.261^2^
Missing	9	0	9	
Mean (SD)	26.3 (16.0)	27.6 (15.3)	26.6 (15.9)	
APOE e4 carrier, *n* (%)	505 (60.5%)	120 (53.8%)	625 (59.1%)	0.072^1^
Baseline SUVr value, Mean (SD)	1.3 (0.2)	1.3 (0.2)	1.3 (0.2)	0.064^2^

^1^ Pearson’s Chi-Squared Test

^2^ Two Sample T-Test

Screening rates on average were higher in non-academic (mean *n* [sd]: 3.5 [3.8]) than academic (mean *n* [sd]: 2.2 [1.3]) sites ( *p* = 0.226), while randomization rates were higher in academic (mean % [sd]: 20.4 [9.2]) than non-academic (mean % [sd]: 17.8 [7.8]) sites (*p* = 0.325) (Table 2); however, the results were not statistically significant.

**Table 2:** Differences in baseline site-level characteristics by site type and the corresponding *p*-values

	Academic (*N* = 835)	Non-academic (*N* = 223)	Total (*N* = 1058)	Unadj. *P* Value
**PI Medical Specialty**				0.338^1^
Missing	4	1	5	
Neurology	33 (71.7%)	8 (57.1%)	41 (68.3%)	
Other	13 (28.3%)	6 (42.9%)	19 (31.7%)	
**Screening Rate**, Mean (SD)	2.2 (1.3)	3.5 (3.8)	2.5 (2.2)	0.226^2^
**Randomization Rate**, Mean (SD)	20.4 (9.2)	17.8 (7.8)	19.8 (8.9)	0.325^2^

^1^ Fisher’s Exact Test

^2^ Two Sample T-Test

This table displays site-level characteristics for 50 academic and 15 non-academic sites

*Recruitment sources*. There were differences in recruitment sources among the site types (Table 3). A higher proportion of participants from academic compared to non-academic sites reported ‘earned media’ (academic: 41.0%; non-academic: 27.5%; adjusted *p* < 0.001) and ‘organizational referral’ (academic: 11.9%; non-academic: 5.4%; adjusted *p* = 0.011) as a source, while a higher proportion of participants from non-academic sites reported ‘advertising’ (academic: 5.7%; non-academic: 13.1%; adjusted *p* < 0.001) and ‘internal referral’ (academic: 39.2%; non-academic: 50.9%; adjusted *p* = 0.005). No difference was observed in the proportion of participants selecting ‘outside physician’ between the site types (academic: 3.9%; non-academic 4.1%; adjusted *p* = 0.900).

**Table 3:** Recruitment source by site type

	Academic (*N* = 827)	Non-academic (*N* = 222)	Total (*N* = 1049)	Unadj. *P* Value	Adj. *P* Value*
**Advertising**, Yes	47 (5.7%)	29 (13.1%)	76 (7.2%)	< 0.001^1^	< 0.001
**Earned Media**, Yes	339 (41.0%)	61 (27.5%)	400 (38.1%)	< 0.001^1^	< 0.001
**Internal Referral**, Yes	324 (39.2%)	113 (50.9%)	437 (41.7%)	0.002^1^	0.005
**Organizational Referral**, Yes	98 (11.9%)	12 (5.4%)	110 (10.5%)	0.005^1^	0.011
**Outside Physician**, Yes	32 (3.9%)	9 (4.1%)	41 (3.9%)	0.9^1^	0.9

Note: Participants had the option of selecting more than one recruitment source. Participants that did not select any recruitment source were not included in this table

^1^ Pearson’s Chi-Squared Test

^*^ Holm’s adjusted *p* value

**Fig. 1:**
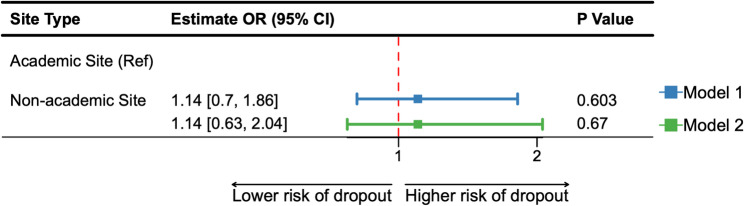
Estimated conditional odds ratio with corresponding 95% confidence interval (CI) and *p*-value. Model 1 represents the results from a multivariate logistic mixed-effects model adjusting for age at consent, family history, APOE e4 carrier status, and baseline STAI. Model 2 represents the results from a multivariate logistic mixed effects model adjusting for age at consent, family history of dementia, APOEe4 carrier status, baseline STAI, race and ethnicity URG, study partner type, education group, diabetes medical history, baseline SUVr category, baseline CDR sum of boxes category and site geographical region

*Dropout and reasons of early discontinuation*. Among randomized participants, 70.9% at academic and 67.7% at non-academic sites completed the blinded phase of the trial. We found no significant differences in time-to-dropout between site types based on the log-rank test (*p* = 0.108) (Supplemental Material 1). As prespecified, we proceeded with a multivariable logistic mixed-effect regression analysis. This subsequent analysis also showed no significant differences between site types (Model 1: OR = 1.14; CI: 0.70, 1.86; *p* = 0.603) (Fig. 1). There were, however, differences in the reasons for dropout (Table 4; *p* = 0.004). Withdrawal or lost to follow-up was the most frequently reported reason for early discontinuation across both site types, but a higher proportion of participants cited this reason in non-academic (56 out of 72 participants who discontinued) compared to academic (140 out of 243 participants who discontinued) sites. A higher proportion of participants at academic sites reported adverse events (academic: 28.8% [70 out of 243]; non-academic: 11.1% [8 out of 72]) and other reasons for dropout (academic: 13.6% [33 out of 243]; non-academic: 11.1% [8 out of 72]). The results from our exploratory descriptive analyses are summarized in Supplemental Material 2 and 3. Among those who voluntarily withdrew or were lost to follow-up, we observed a higher proportion of females in non-academic than academic sites. No other trends emerged.

**Table 4:** Primary reasons of early study discontinuation by site type

	Academic (*N* = 243)	Non-academic (*N* = 72)	Total (*N* = 315)	*P* Value
**Primary Reasons of Study Discontinuation**				0.004^1^
Withdrawal or lost to follow-up	140 (57.6%)	56 (77.8%)	196 (62.2%)	
Adverse events	70 (28.8%)	8 (11.1%)	78 (24.8%)	
Other	33 (13.6%)	8 (11.1%)	41 (13.0%)	

^1^ Pearson’s Chi-Squared Test

## Discussion

Preclinical AD trials are likely to become more prominent in AD drug development [17, 18]. The A4 trial was among the first multisite preclinical AD trials and presents a unique opportunity to learn about the impact of site type on trial outcomes and instruct future study design choices and conduct in these trials. We observed some meaningful differences between site type in participants’ baseline characteristics, recruitment sources, and reasons for discontinuation that trial investigators may consider during the early planning stages. We did not, however, observe differences in retention rates between academic and non-academic sites.

Consistent with previous literature [15, 19], participants recruited to academic and non-academic sites in the A4 study differed in some demographic and other characteristics. Education levels were high in both site types compared to the average United States population of older adults, but this was even more pronounced for academic sites [20]. The relatively higher education levels in academic sites were also reported in the earlier MCI trial [15] and are in line with other academic studies [21] and recruitment registries [22–24]. Participants at academic sites were also more likely to have a family history of dementia. Participants aware of their predisposition to AD may have existing relationships with memory clinics and be more inclined to seek participation in AD studies through research-focused academic institutions [25]. Non-academic sites on the other hand recruited a higher proportion of participants with non-spousal study partners, diabetes history, CDR-SB score above 0, and belonging to a RE-URG. These findings may suggest that non-academic sites are located in areas more accessible to participants from their local community who may be more representative of the general population. While further investigation is needed to understand why CDR-SB scores differed by site type, it is possible that the potential differences in participant characteristics between site types may have contributed to the higher scores in non-academic (CDR-SB score above 0: 23%) compared to academic sites (CDR-SB score above 0: 7%). The CDR-SB was used as one of the secondary endpoints in the A4 study and is a widely accepted outcome measure for AD trials [26]. This underscores the need for additional research on CDR-SB scores in cognitively unimpaired individuals and to determine if baseline differences impact long-term outcomes in trial settings. It should be noted that all participants were required to be cognitively unimpaired to meet study eligibility and that these CDR-SB score differences were observed despite the similarities in Alzheimer’s Disease Cooperative Study Preclinical Alzheimer’s Cognitive Composite (PACC) and cognitive function index (CFI) scores between site types. When examining site level characteristics, we found that non-academic sites had a higher screening rate (number of participants screened/site/month), but a lower randomization rate (randomized/screened) compared to academic sites, although the differences were not statistically significant. Lower rates of ineligibility reduce cost and expedite trial completion. Previous studies, however, suggest that historically underrepresented groups may be less likely to qualify for trials [27], perhaps indicating that maximizing the representativeness of trial samples requires investments in broad recruitment approaches with wide reach and potential greater rates of ineligibility. These results suggest the importance of having non-academic sites to achieve this goal of a broad recruitment funnel to reach historically underrepresented populations who might not have otherwise had access to trials.

Previous analysis of A4 data showed significant differences in the frequency of recruitment sources by race and ethnicity [27], and in the current study, we observed substantial differences in recruitment sources by site type. Academic sites in the A4 trial relied heavily on national and local media (41%) for broader public visibility to recruit eligible participants. Conversely in non-academic sites, more than 50% of the randomized participants were recruited through internal referral.

Significant differences by site type in participant characteristics did not translate to differences in retention rates in our study. There are several factors that may account for the discrepancy between our findings and the results from the MCI trial reported by Edland et al. (2010). Most notably, the two studies [10, 28], recruited samples of strikingly different disease severities and were conducted over a decade apart. Although participants diagnosed with MCI are functionally unimpaired, they experience impairments in memory and other cognitive domains. Participants in preclinical AD trials on the other hand are asymptomatic. Participants in MCI trials may be more reliant on external factors, such as their study partners, in continuing participation and completing the trial. Similar to our findings, no site type differences in retention rates were observed in a trial for Major Depressive Disorder, where the participants were cognitively unimpaired [29].

When examining reasons for discontinuation, we found a higher proportion of participants who withdrew or were lost to follow up in non-academic than academic sites. Site differences may create other challenges not captured by our data. For example, academic sites may be more likely to have imaging, laboratory, and other necessary capabilities in a single location, lowering site burden when starting a trial and simplifying scheduling and attending study visits for participants. Differences by site type in participant characteristics (having comorbidities, enrolling with non-spousal study partners and belonging to more underserved populations) may have also contributed to these differences. In a previous analysis of the A4 data, a notable trend was observed in the association between partner type and dropout, though the results were not statistically significant [16]. Non-spousal informants largely consist of working family members and friends who may find the long trial duration and frequent study visits challenging to sustain. The decision to voluntarily dropout of the trial may also reflect participant and/or study partner expectations or experiences with the trial. Future studies should consider examining potential site type differences in participant feedback as they may be instrumental in understanding dyad decisions around ongoing participation.

There were limitations to our study. Retrospective analyses such as those included in this study generate novel hypotheses but cannot determine the direct causal effects. The A4 trial experienced a hiatus in testing and dose administration due to the Covid-19 pandemic, though only 10 participants reported this disruption as their primary reason of early study discontinuation. The A4 trial had 15 participating non-academic sites, which represents less than one third of all sites. As a result, some of the analyses may be underpowered to detect site type differences. While all trials suffer from participation bias, the A4 trial had a small sample of participants identifying as having lower education or belonging to an underrepresented racial or ethnic group. We previously demonstrated that individuals from these groups were less likely to be eligible for the A4 study. Specifically, clinical, cognitive, and biomarker enrollment criteria all produced statistically significant differential eligibility rates among study subpopulations [27]. Ongoing preclinical AD trials, such as the AHEAD 3–45 Study [30], will address some of these limitations through systematic data collection related to participant characteristics, recruitment sources, and retention outcomes, allowing us to expand on our findings with greater granularity when they are completed.

## Conclusions

Differences by site type in recruitment sources as well as baseline participant characteristics did not translate to significant differences in retention rates in the A4 trial. While the results did not reach statistical significance, we observed that non-academic sites had a higher screening rate, but a lower randomization rate compared to academic sites. Distinctions between academic and non-academic sites in reasons for discontinuation were observed, with higher proportion of voluntary withdrawal or lost to follow up in non-academic sites. Ongoing and future trials will allow us to perform more in-depth investigation to elucidate and confirm these results.

## Supplementary Information

Below is the link to the electronic supplementary material.

Supplementary Material 1

## Data Availability

The A4 and LEARN Studies were coordinated by ATRI at the University of Southern California, and the data are made available through the A4 website (https://www.a4studydata.org).

## Abbreviations

A4: Anti-Amyloid Treatment in Asymptomatic Alzheimer’s disease
AARP: American Association of Retired Persons
AD: Alzheimer’s Disease
ADCS PACC: Alzheimer’s Disease Cooperative Study Preclinical Alzheimer’s Cognitive Composite
ADRC: Alzheimer’s Disease Research Center
ATRI: Alzheimer’s Therapeutic Research Institute
CDR-SB: Clinical Dementia Rating – Sum of Boxes
CFI: Cognitive Function Index
CI: Confidence Interval
MCI: Mild Cognitive Impairment
NIH: National Institutes of Health
OR: Odds Ratio
PET: Positron Emission Tomography
STAI: State-Trait Anxiety Inventory
