# Supplementary material for: Recruitment and retention in a preclinical AD trial: comparisons between academic and non-academic sites
Source: Alzheimers Res Ther. 2025 Oct 14;17:222. doi: 10.1186/s13195-025-01867-8 (PMC12522582; doi:10.1186/s13195-025-01867-8)
Supplement: Supplementary file 1 — Supplementary Material 1 [file 13195_2025_1867_MOESM1_ESM.docx]

Supplemental Material 1. Kaplan-Meier plot on time to study discontinuation by site type and log-rank test


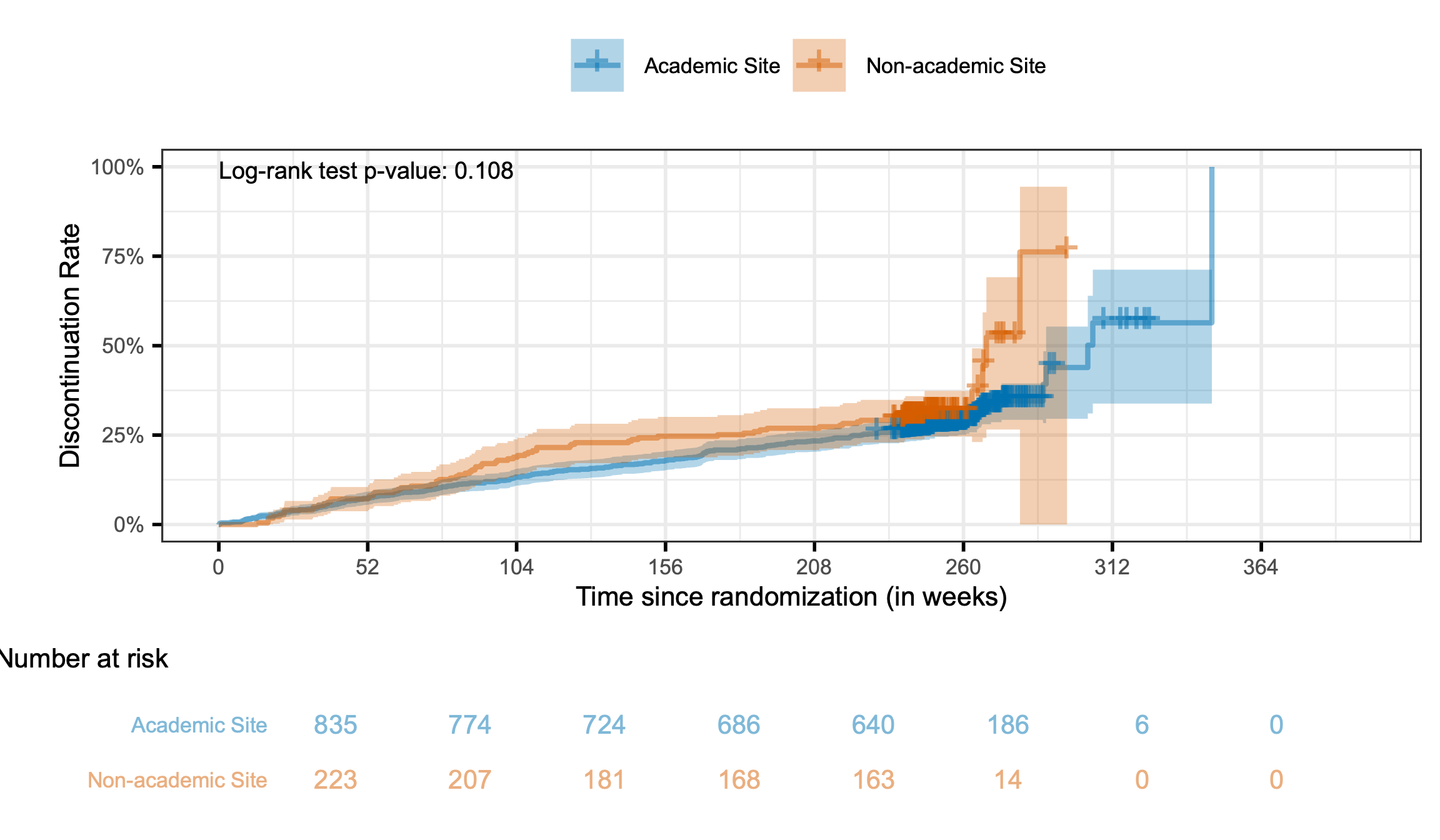


Supplemental Material 2. Summary of adverse events by site type

| Reasons | System Organ Class | Site Type, n (%) | |
| --- | --- | --- | --- |
|  |  | Academic | Non-academic |
| Death | - | 8 (11.4) | 2 (25.0) |
| Safety risk | - | 7 (10.0) | 2 (25.0) |
| Adverse event | - | 1 (1.4) | 0 (0.0) |
|  | Cardiac disorders | 4 (5.7) | 1 (12.5) |
|  | General disorders and administration site conditions | 1 (1.4) | 0 (0.0) |
|  | Immune system disorders | 1 (1.4) | 0 (0.0) |
|  | Infections and infestations | 2 (2.9) | 0 (0.0) |
|  | Injury, poisoning and procedural complications | 2 (2.9) | 0 (0.0) |
|  | Investigations | 1 (1.4) | 0 (0.0) |
|  | Musculoskeletal and connective tissue disorders | 2 (2.9) | 0 (0.0) |
|  | Neoplasms benign, malignant and unspecified (incl cysts and polyps) | 18 (25.7) | 0 (0.0) |
|  | Nervous system disorders | 12 (17.1) | 2 (25.0) |
|  | Respiratory, thoracic and mediastinal disorders | 1 (1.4) | 0 (0.0) |
|  | Skin and subcutaneous tissue disorders | 6 (8.6) | 0 (0.0) |
|  | Vascular disorders | 4 (5.7) | 0 (0.0) |
|  | Hepatobiliary disorders | 0 (0.0) | 1 (12.5) |
| Total | - | 70 | 8 |

Supplemental Material 3. Site type differences in baseline characteristics of those who prematurely discontinued the trial due to voluntary withdrawal or lost to follow-up

|  | Academic Site (N=140) | Non-academic Site (N=56) | Total (N=196) |
| --- | --- | --- | --- |
| **Sex** |  |  |  |
| Male | 57 (40.7%) | 13 (23.2%) | 70 (35.7%) |
| Female | 83 (59.3%) | 43 (76.8%) | 126 (64.3%) |
| **Age at Consent** |  |  |  |
| Mean (SD) | 72.1 (5.2) | 72.4 (5.4) | 72.2 (5.3) |
| Median | 70.9 | 72.3 | 71.2 |
| Range | 65.0 - 85.2 | 65.1 - 85.0 | 65.0 - 85.2 |
| **Race** |  |  |  |
| Missing | 1 | 0 | 1 |
| American Indian or Alaskan Native | 0 (0.0%) | 1 (1.8%) | 1 (0.5%) |
| Asian | 2 (1.4%) | 0 (0.0%) | 2 (1.0%) |
| Black or African American | 7 (5.0%) | 4 (7.1%) | 11 (5.6%) |
| Native Hawaiian or Other Pacific Islander | 0 (0.0%) | 0 (0.0%) | 0 (0.0%) |
| More than one race | 1 (0.7%) | 1 (1.8%) | 2 (1.0%) |
| White | 129 (92.8%) | 50 (89.3%) | 179 (91.8%) |
| **Ethnicity** |  |  |  |
| Hispanic or Latino | 2 (1.4%) | 5 (8.9%) | 7 (3.6%) |
| Not Hispanic or Latino | 138 (98.6%) | 51 (91.1%) | 189 (96.4%) |
| **Race and Ethnicity URG** |  |  |  |
| Not URG | 128 (91.4%) | 46 (82.1%) | 174 (88.8%) |
| URG | 12 (8.6%) | 10 (17.9%) | 22 (11.2%) |
| **Race and Ethnicity/Education URG** |  |  |  |
| Not URG | 118 (84.3%) | 42 (75.0%) | 160 (81.6%) |
| URG | 22 (15.7%) | 14 (25.0%) | 36 (18.4%) |
| **Marital Status** |  |  |  |
| Divorced | 25 (17.9%) | 11 (19.6%) | 36 (18.4%) |
| Never married | 10 (7.1%) | 2 (3.6%) | 12 (6.1%) |
| Widowed | 11 (7.9%) | 5 (8.9%) | 16 (8.2%) |
| Married | 94 (67.1%) | 38 (67.9%) | 132 (67.3%) |
| **Study Partner Type** |  |  |  |
| Missing | 1 | 0 | 1 |
| Spouse | 83 (59.7%) | 33 (58.9%) | 116 (59.5%) |
| Adult Child/Child-in-law | 22 (15.8%) | 4 (7.1%) | 26 (13.3%) |
| Other | 34 (24.5%) | 19 (33.9%) | 53 (27.2%) |
| **Family Dementia History** |  |  |  |
| No | 44 (31.4%) | 19 (33.9%) | 63 (32.1%) |
| Yes | 96 (68.6%) | 37 (66.1%) | 133 (67.9%) |
| **Education** |  |  |  |
| Mean (SD) | 16.8 (2.8) | 16.1 (3.2) | 16.6 (2.9) |
| Median | 17.5 | 16.0 | 16.0 |
| Range | 8.0 - 25.0 | 9.0 - 30.0 | 8.0 - 30.0 |
| **Education Category** |  |  |  |
| High School Graduate and Below | 11 (7.9%) | 5 (8.9%) | 16 (8.2%) |
| Some College Degree | 59 (42.1%) | 34 (60.7%) | 93 (47.4%) |
| Professional Degree | 70 (50.0%) | 17 (30.4%) | 87 (44.4%) |
| **Treatment Group** |  |  |  |
| Placebo | 63 (45.0%) | 29 (51.8%) | 92 (46.9%) |
| Solanezumab | 77 (55.0%) | 27 (48.2%) | 104 (53.1%) |

† A response ‘Unknown or Not Reported‘ for race or ethnicity were considered as missing values in this analysis.
